# A comprehensive study on microstructure and tensile behaviour of a selectively laser melted stainless steel

**DOI:** 10.1038/s41598-018-26136-7

**Published:** 2018-05-17

**Authors:** Chunlei Qiu, Mohammed Al Kindi, Aiman Salim Aladawi, Issa Al Hatmi

**Affiliations:** 1 School of Materials Science and Engineering, Beihang University, Beijing, 100191 China; 2 School of Engineering, Cardiff University, The Parade, Cardiff, CF24 3AA UK

## Abstract

316L stainless steel samples have been prepared by selective laser melting (SLM) using a pulsed laser mode and different laser powers and scanning patterns. The as-fabricated samples were found to be dominated by clusters of nano-sized *γ* needles or cells. TEM imaging shows that these needles contain a high population of dislocations while TEM-EDX analysis reveals high chemical homogeneity throughout the as-fabricated samples as evidenced by the fact that there is even no micro-/nano-segregation at interfaces between neighbouring *γ* needles. The good chemical homogeneity is attributed to the extremely high cooling rate after SLM (>10^6^ °C/s) and the formation of Si- and Mn-oxides that distribute randomly in the current samples. The laser-processed samples show both superior strength and ductility as compared with conventionally manufactured counterparts. TEM examination on the deformed specimens reveals a significantly high density of dislocations and a great number of twinning within nano-needles, suggesting that the plastic deformation has been governed by both gliding of dislocations and twinning deformation, which is believed to be responsible for the simultaneous acquisition of superior strength and ductility. Finally, laser power shows a much more dominant role than laser scanning pattern in porosity and grain size development for the SLM-processed 316L stainless steel samples.

## Introduction

Additive manufacturing such as selective laser melting (SLM) nowadays has been widely used to process various metallic materials and fabricate parts for various applications due to their excellent net-shape manufacturing capacity. These technologies, however, also show very unique processing characteristics that can significantly affect structural integrity and microstructure of a material. For example, complex laser-powder interaction can lead to formation of pores within as-fabricated materials^1–4^. Great thermal gradient between melt pools and substrate/previous layers can result in development of strong texture and columnar grains in built samples. High cooling rate leads to non-equilibrium microstructures that are very different from those formed by other methods such as casting or forging^5^. For many materials, rapid solidification and cooling during SLM successfully suppress the development of dendrites and instead very fine cellular structure with cell width that could be only several hundred nanometers is developed. The formation of this kind of cellular structure is usually associated with micro-/nano-segregation at cell walls. For example, Loretto *et al*.^6,7^ revealed that the nano-sized cellular structure within SLM-processed AlSi10Mg is associated with segregation of Si at cell walls and the cellular structure in SLM-processed CM247LC is due to development of *γ*′/*γ* eutectic and segregation of Hf/Ti/Ta/W at cell walls. Cellular structure in SLM-processed stainless steels such as 316L stainless steel has also been reported in a number of studies^8–14^ and was found to be associated mainly with segregation of Cr and Mo at cell walls^12–14^. It is believed that the elemental segregation at cell walls is due to solute redistribution during solidification^6,7^. Loretto *et al*.^7^ concluded that the segregation of Hf, Ti, Ta and W at cell walls in SLM-processed CM247LC is a result of partitioning of these elements into the liquid together with Ni, Co and W partitioning into the solid during solidification after SLM. The solute redistribution is, however, greatly affected by solidification/cooling rate^15–18^. Studies on solidification behavior of stainless steels with low Cr_eq_/Ni_eq_ ratio suggest that high cooling rates led to a reduction in the amount of solute redistribution during solidification and consequently to a decrease in ferrite content^15–18^. Previous work^19–24^ also show that at low solidification rate (i.e., solid-liquid interface growth velocity), solute redistribution experiences a transition from a segregation at low solidification rate to a solute trapping effect by a growing solid in rapid solidification, i.e., at high solid-liquid interface growth velocities, solute concentration in solids could exceed conventional solid solubility limits given by the equilibrium phase diagram. With stronger solute trapping effect, less microsegregation would be expected. Given that SLM is a process that involves rapid solidification and cooling, it is not clear to what extent the solute redistribution can be supressed and whether a solute trapping effect can be achieved during this process. In this study, SLM with a pulsed laser mode is used to produce 316L stainless steel samples for an investigation on chemical distribution from macroscopic level down to nanometer level with the aim of developing a better understanding on the solidification behavior and microstructural development during SLM. The use of a pulsed laser mode is to generate more discrete molten pools and thus higher cooling rates during solidification as compared with continuous laser mode that has been used in the majority of previous studies. EDX in both SEM and TEM will be used to investigate the chemical distribution and homogeneity within the SLM-processed 316L samples.

Moreover, it is noted that additively manufactured metallic materials such as Ti-6Al-4V, Ni-based superalloys and Al-Si alloys usually demonstrate high strengths but relatively poor elongations either due to presence of defects or residual stress or brittle fracture mode^6,7,25,26^. Additively manufactured 316L stainless steel, however, is among some of the rare additively manufactured metallic materials that show both high strengths and elongations despite the presence of porosity^8–14^. This is obviously associated with the microstructure and deformation mechanism of this material. However, the reports on deformation and strengthening mechanisms of additively manufactured 316L stainless steel are limited. Only till very recently, Pham *et al*.^27^ reported that deformation twinning is active during the plastic deformation of SLM-processed 316L stainless steel through EBSD study and is believed to be responsible for the excellent ductility of the material. This was also confirmed by Wang *et al*.’s work through TEM study^12^. While the role of twinning deformation is recognized in contributing to good ductility and strengths in these studies, the investigation on the dislocation structure of tensile deformed SLM-processed 316L stainless steel is lacking. To comprehensively understand the deformation mechanisms of SLM-processed 316L stainless steel, it is necessary to conduct an in-depth investigation into the microstructure and dislocation structure before and after tensile deformation. In this study, the SLM-processed 316L samples will be extensively investigated using TEM before and after tensile testing to develop a better understanding on deformation and strengthening mechanisms of the SLM-processed 316L stainless steel.

The third aim of this paper is to investigate the dominant factor between two categories of processing parameters (laser parameters such as laser power and scanning speed, and laser scanning patterns) that affects porosity and microstructural development most. This is particularly important to industrial production as extensive parametric study is time-consuming and labor-intensive and is not feasible in a large-scale manufacturing environment. In this study, 316L samples will be fabricated at different laser powers and with different laser scanning patterns. The as-fabricated samples will be investigated in terms of their porosity and grain structure.

## Results

### Study on porosity

**Figure 1:**
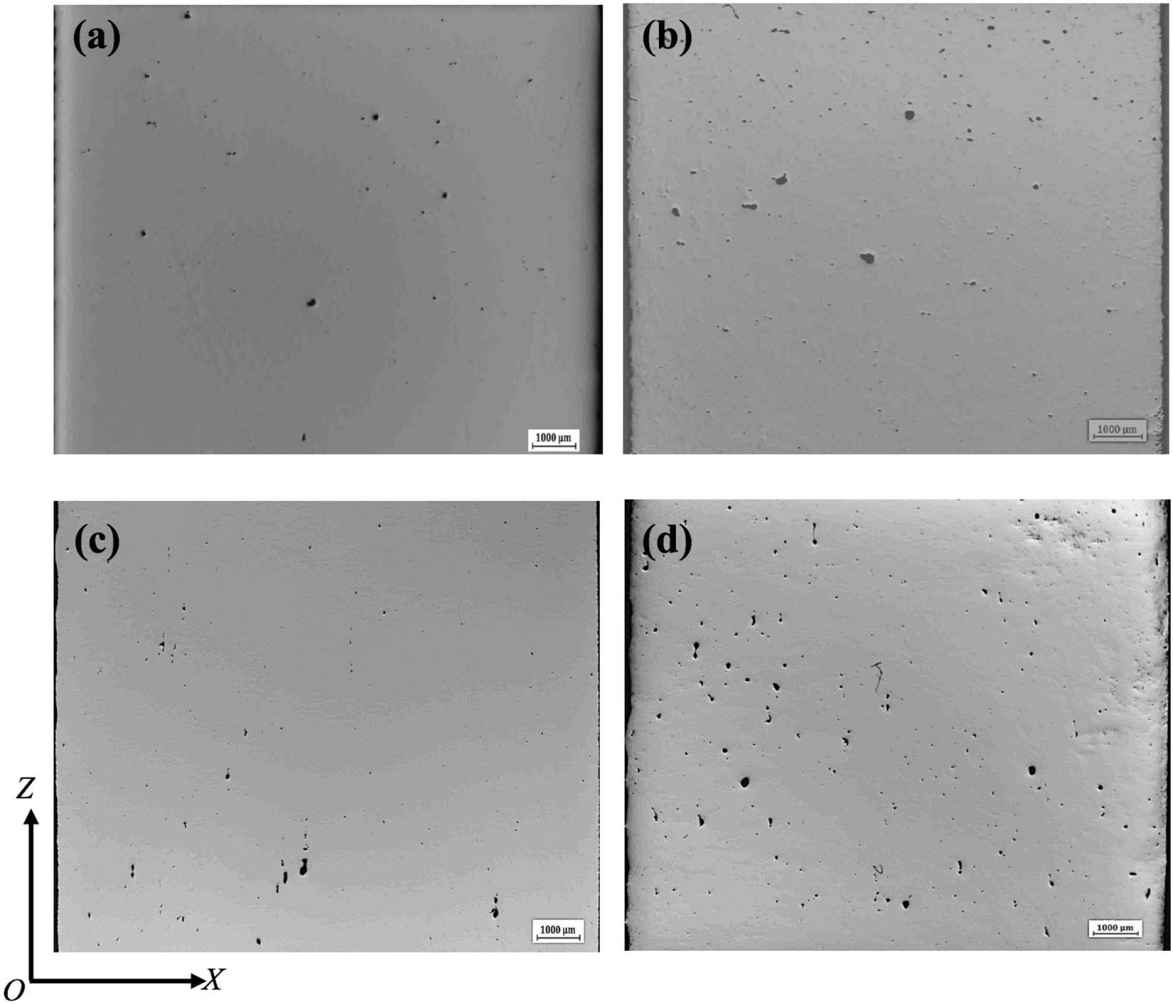
Distribution of pores within the samples fabricated at 200 W with various laser scanning strategies, (**a**) Meander, A_f_ = 0.14%; (**b**) Stripe, A_f_ = 0.42%; (**c**) Chessboard with 5 × 5 mm islands, A_f_ = 0.19%; (**d**) Chessboard with 1 × 1 mm islands, A_f_ = 0.61%.

**Figure 2:**
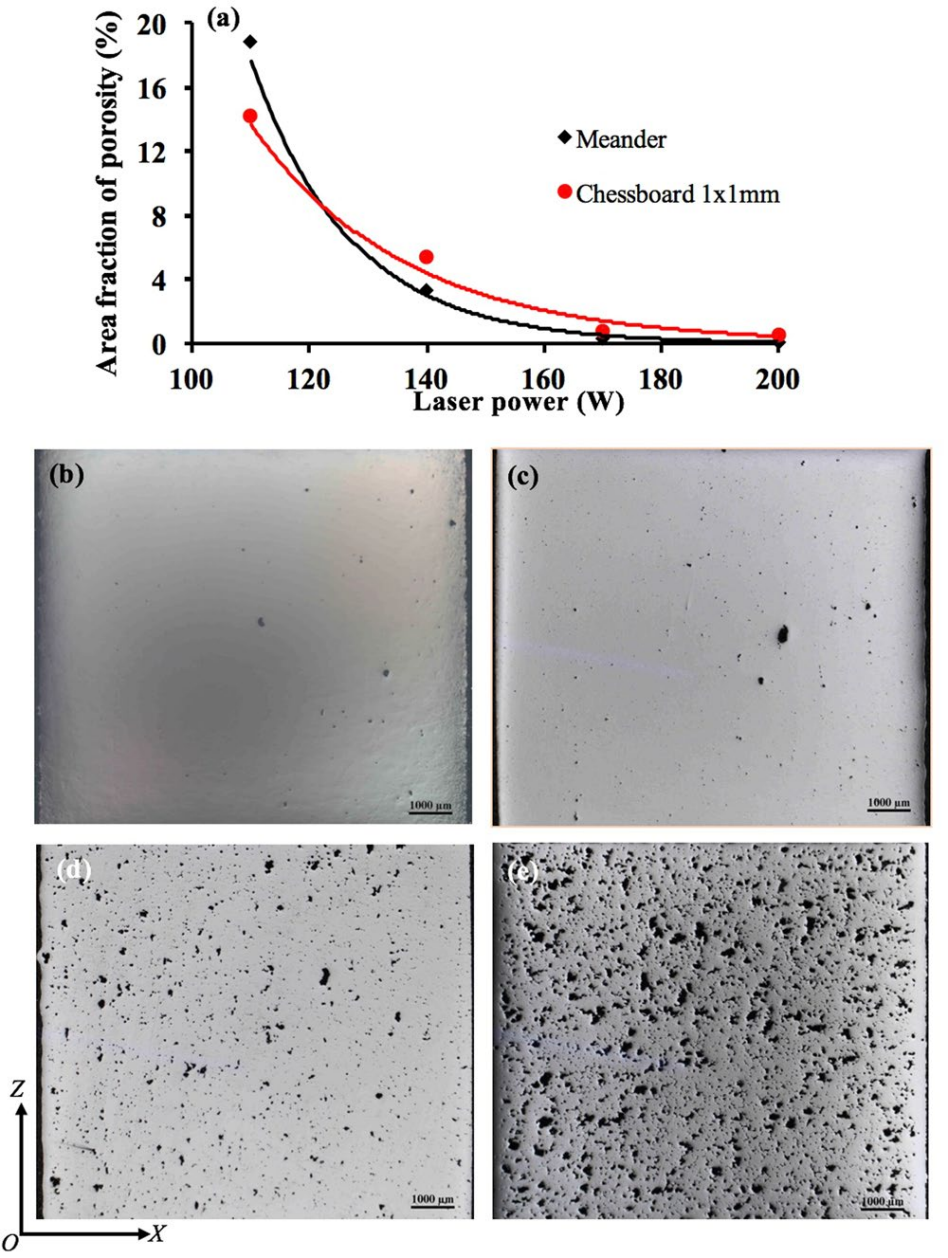
(**a**) A plot showing the variation of porosity level with laser power, (**b**) 200 W, A_f_ = 0.14%; (**c**) 170 W, A_f_ = 0.35%; (**d**) 140 W, A_f_ = 3.3%; (**e**) 110 W, A_f_ = 18.9%. The samples shown in (**b**–**e**) were made with Meander scanning strategy. The scatter of porosity level is within ± 0.2%.

Figure 1 shows the distribution of pores within 316L samples fabricated under pulsed laser mode and at 200 W with different laser scanning strategies. It is obvious that the laser scanning strategy shows no significant influence on porosity or structural integrity of the laser processed samples. Actually, the samples fabricated using different laser scanning strategies show highly comparable porosity levels. The sample fabricated with Chessboard scanning strategy with island size of 1 × 1 mm seems to show the highest porosity level among all the samples but its porosity level of 0.61% is still very low. In contrast to laser scanning strategy, laser power obviously shows much more pronounced influence on porosity development, as shown in Fig. 2. It can be seen that the porosity level of samples made with either Meander or Chessboard strategy has decreased exponentially with increased laser power (from 110 W to 200 W). The size of pores increases continuously with decreased laser power and their morphology becomes increasingly irregular-shaped, changing from spherical to more elongated or polygonal shape. The results suggest that laser power is a more determining factor in porosity development of 316L sample than laser scanning strategy during SLM.

### Microstructural characterisation

**Figure 3:**
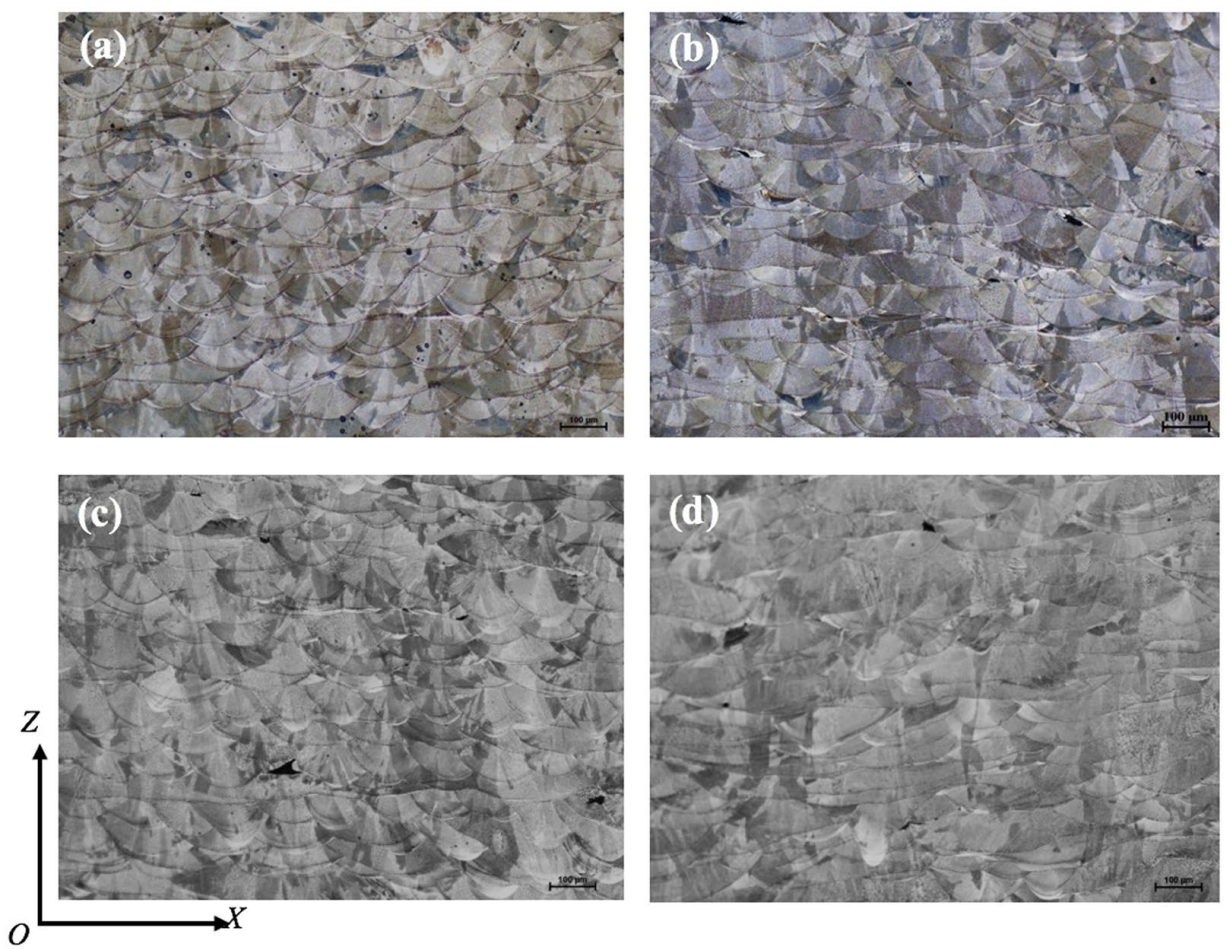
OM micrographs showing the microstructure of 316L samples fabricated at 200 W using different laser scanning strategies, (**a**) Meander; (**b**) Stripe; (**c**) Chessboard with 5 × 5 mm islands; (**d**) Chessboard with 1 × 1 mm islands.

**Figure 4:**
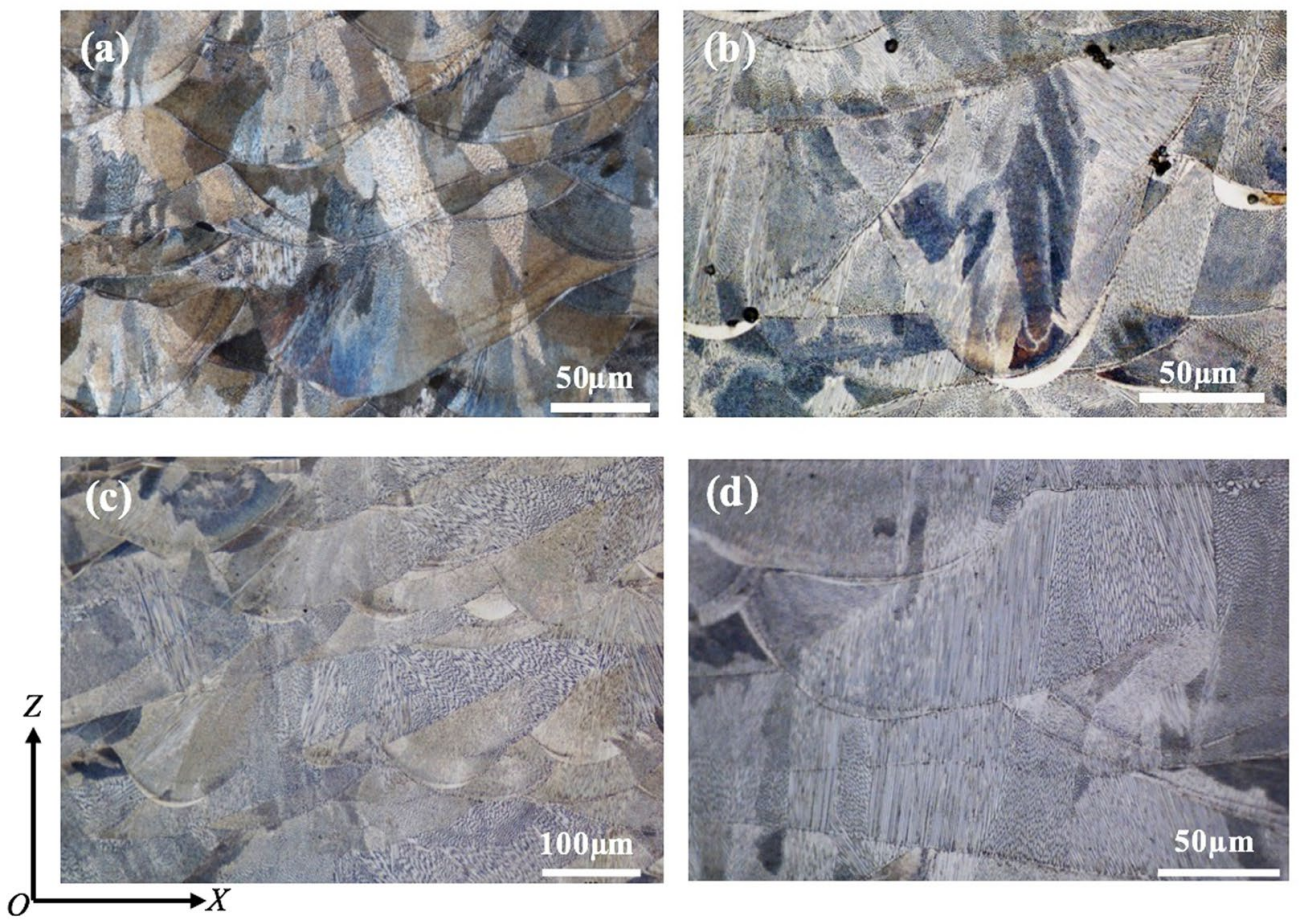
High magnification OM micrographs showing the microstructure of 316L samples fabricated at 200 W using different scanning strategies, (**a**) Meander; (**b**) Stripe; (**c**) Chessboard with 5 × 5 mm islands; (**d**) Chessboard with 1 × 1 mm islands.

**Figure 5:**
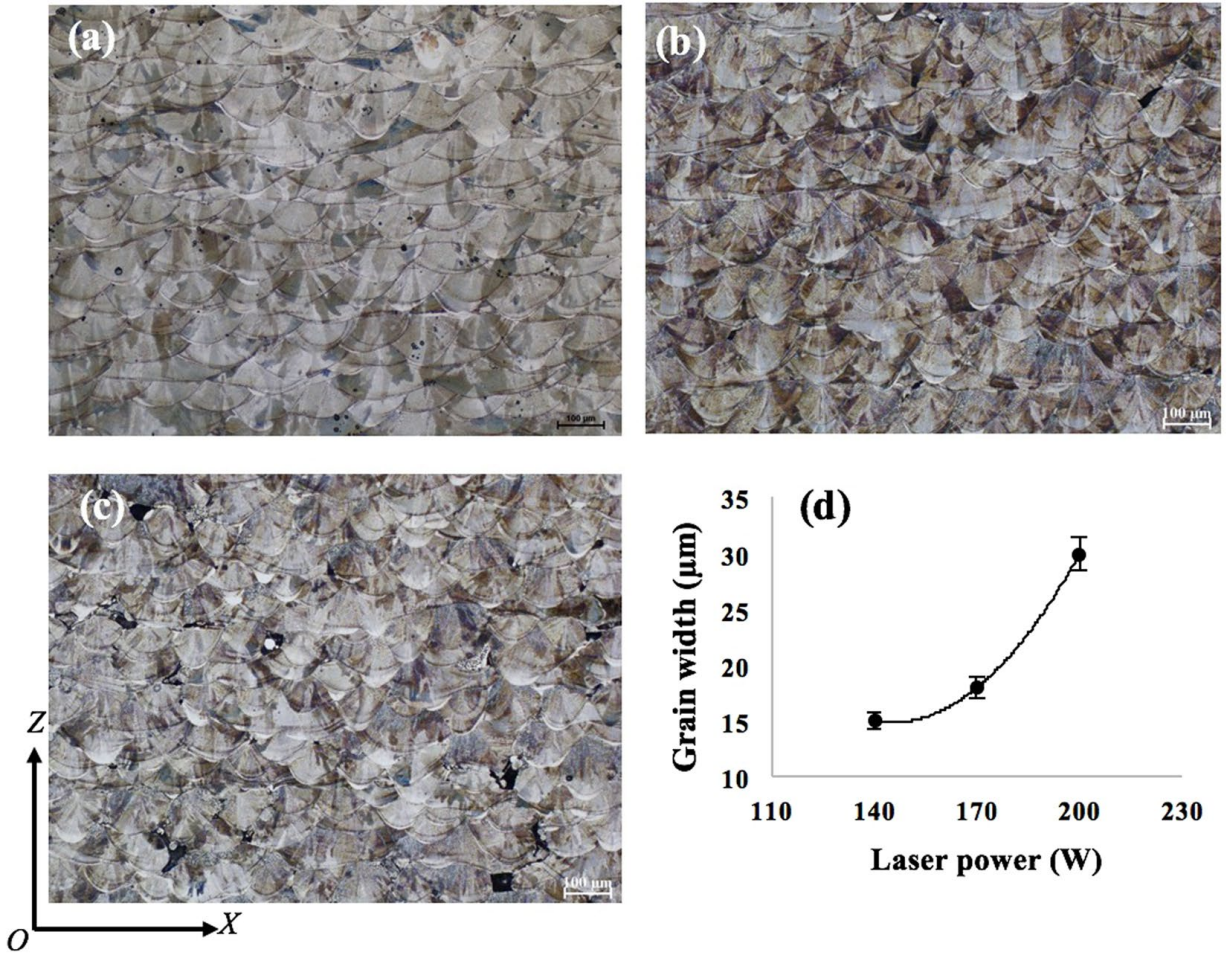
OM micrographs showing the microstructure of 316L samples fabricated with Meander scanning strategy but at different laser powers, (**a**) 200 W; (**b**) 170 W; (**c**) 140 W; (**d**) a plot showing the dependence of grain width on laser power.

**Figure 6:**
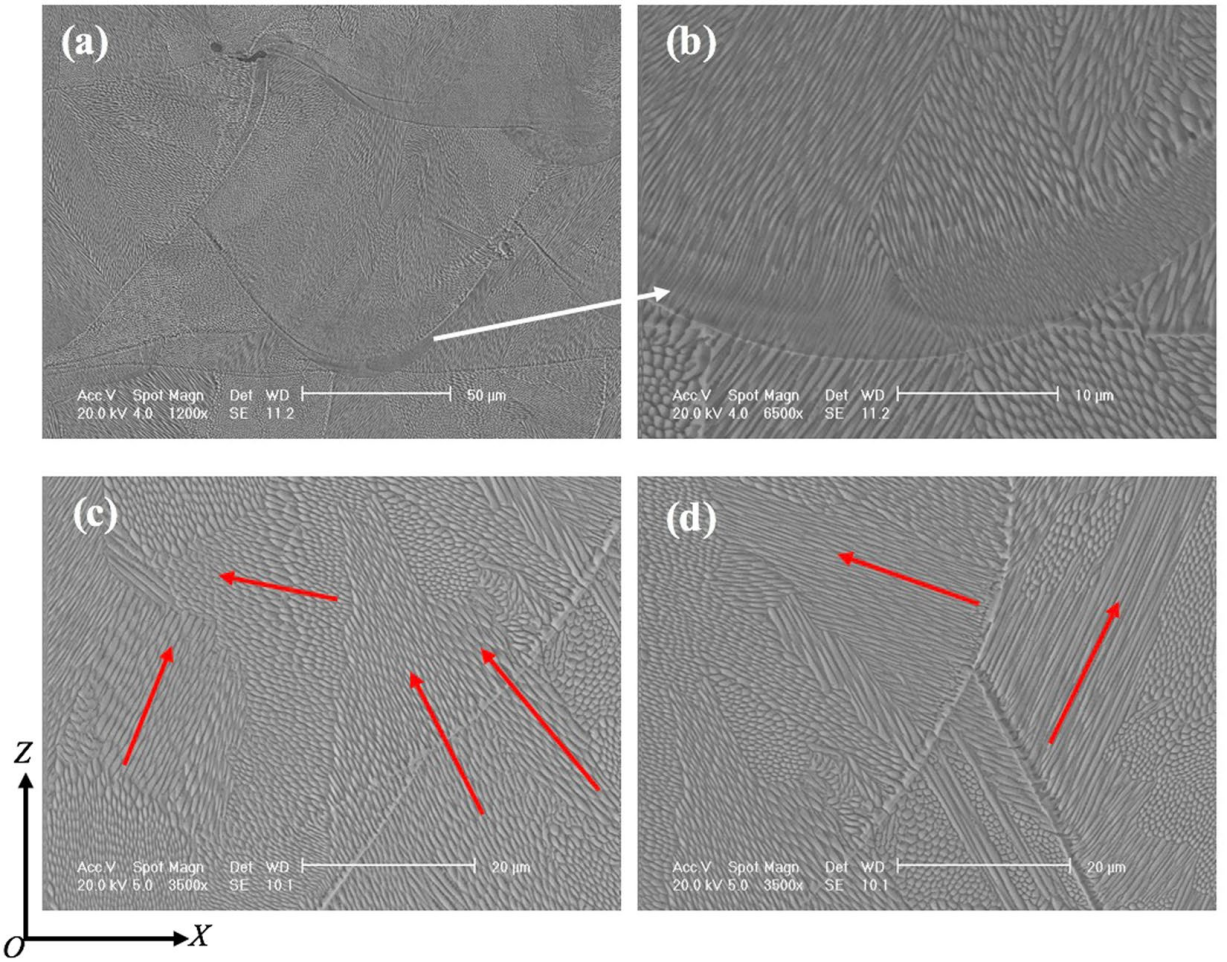
SEM micrographs showing the microstructure of 316L samples after SLM at 200 W with Meander scanning strategy, (**a**) within a weld bead; (**b**) at the bottom region of a single weld bead; (**c**) grain structure beyond two layers; (**d**) grains in two neighbouring weld beads. The arrows show how needle/cellular structures grow in each grain.

Figures 3 and 4 show the microstructure of 316L samples fabricated under pulsed laser mode and at 200 W with different laser scanning strategies. All the samples are dominated by columnar grains. However, these columnar grains are generally contained within a single weld bead or within two neighbouring layers. As compared with previous studies on SLM or DLD of 316L where columnar grains could grow beyond many layers^10^, the epitaxial grain growth in the current study is obviously much less pronounced. It is also noted that there is no obvious difference regarding the grain size among the samples fabricated with different scanning strategies; see Fig. 3. In contrast, laser power shows much more influence on grain structure development. According to Fig. 5, the grain size has continuously decreased with decreased laser power. Each grain in the as-fabricated samples is dominated by a cluster of needle structure or cellular structure which extends along a single orientation; see Fig. 4. This is further confirmed by SEM observation as shown in Fig. 6 which clearly reveals different clusters of needles oriented in different directions. These needles/cells usually have a diameter of 500–800 nm and have very high aspect ratios. Moreover, in all the samples fabricated, a weld bead was generally found to be associated with a relatively bright arc-shape region at the bottom of the bead under OM observation (see Figs 3–5, particularly Fig. 4(b)). SEM examination shows that these regions actually correspond to even finer needles (smaller than 300 nm in diameter); see Fig. 6(b). EDX analysis on these regions and regions from the interior of a weld bead shows no obvious difference in composition; see Table 1. This result suggests that the difference in microstructure between the bottom region and the upper region of a weld bead should not be due to chemical factor but may be due to difference in thermal history (such as cooling rate) in different regions. The finer microstructure at the bottom region of a weld bead is probably due to a higher cooling rate at the interface between the melt pool and previous layers.

**Table 1:** SEM-EDX analysis results showing the compositions in different regions of a solidified melt pool (wt.%).

Element	Bottom region 1	Bottom region 2	Bottom region 3	Upper region 1	Upper region 2	Upper region 3
Si	0.75	0.86	0.8	0.78	0.84	0.83
Cr	18.65	18.41	18.5	18.43	18.39	18.68
Mn	1.23	1.3	1.24	1.21	1.27	1.16
Fe	64.32	64.18	64.48	64.58	64.62	64.43
Ni	12.19	12.05	11.89	12.02	11.98	11.96
Mo	2.85	3.2	3.09	2.99	2.89	2.94

**Figure 7:**
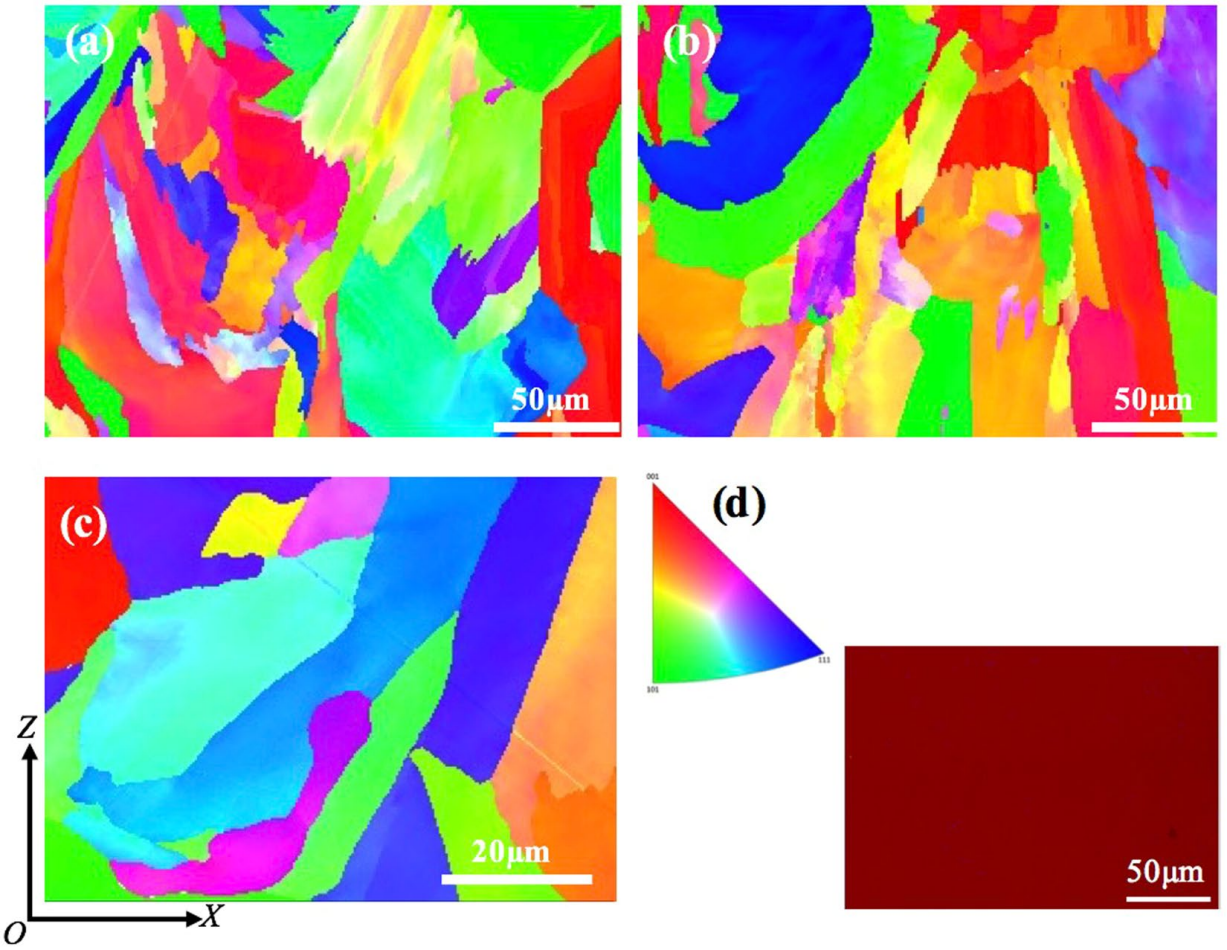
EBSD images showing the grain structure of samples fabricated with different scanning strategies, (**a**) Meander; (**b**,**c**) Chessboard 1 × 1 mm; (**d**) the inverse pole figure for (**a**–**c**) and the phase mapping for (**a**) (the red colour represents γ phase and the blue colour α phase; the black colour corresponds to unknown phases).

**Figure 8:**
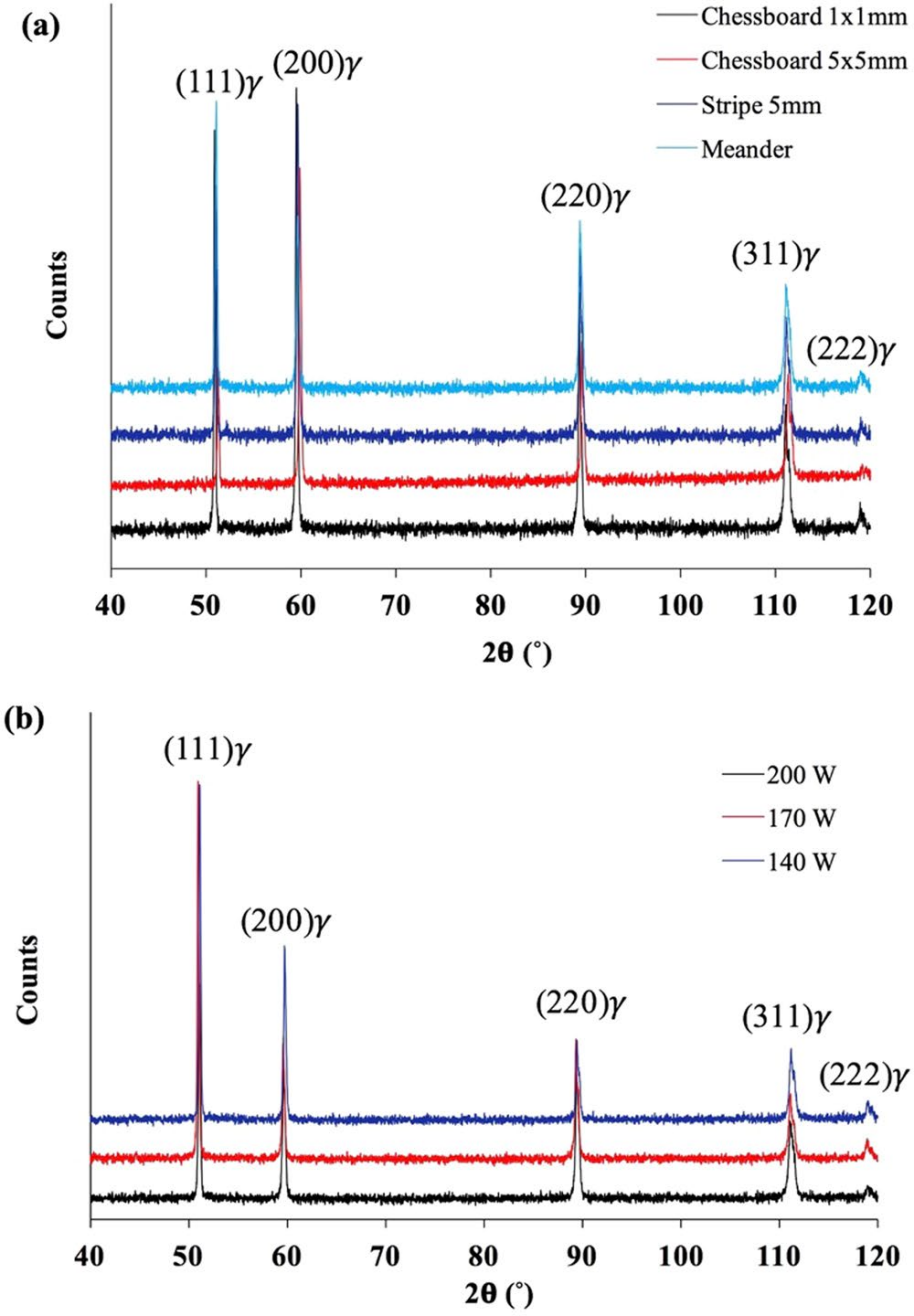
XRD results for samples fabricated using (**a**) different laser scanning strategies at 200 W; (**b**) different laser powers with Meander scanning strategy.

To further investigate the grain structure, particularly grain orientations, EBSD study has been performed on some of the as-fabricated samples. The results are shown in Fig. 7. In agreement with the above SEM observation, EBSD results further confirm that grains in the as-fabricated samples have grown in different orientations and as a result there is no pronounced texture in the current samples. This kind of random grain distribution within individual solidified melt pool suggests that the use of pulsed laser mode in the current study may have yielded a more discrete melt pool during SLM. The influence of laser scanning strategy on the grain structure development is not so obvious either. This result suggests that the thermal history of melt pools during SLM under the current processing conditions was highly localised. Moreover, during EBSD analysis, the phase mapping (not shown here) indicated that more than 99% of the areas analysed are *γ* phase. This is consistent with XRD analysis on the as-fabricated samples as shown in Fig. 8 which confirms that the current SLM-processed 316L samples are all dominated by *γ* phase. Therefore, the nano-needles shown in Fig. 6 are obviously *γ* nano-needles. This result is also consistent with previous studies on the microstructure of SLM-processed 316L^8–14^. In the XRD analysis results, it is also noted that the sample fabricated at 200 W with Chessboard scanning strategy with island size of 1 × 1 mm shows peaks that tend to shift towards lower angles as compared with the rest of the samples. This may be due to that a different level of residual stress has been developed within the sample given that the laser scanning strategy used for this sample is much more dispersive and random than the rest and thus the the thermal history could be kind of different from the other samples.

### Chemical analysis

**Figure 9:**
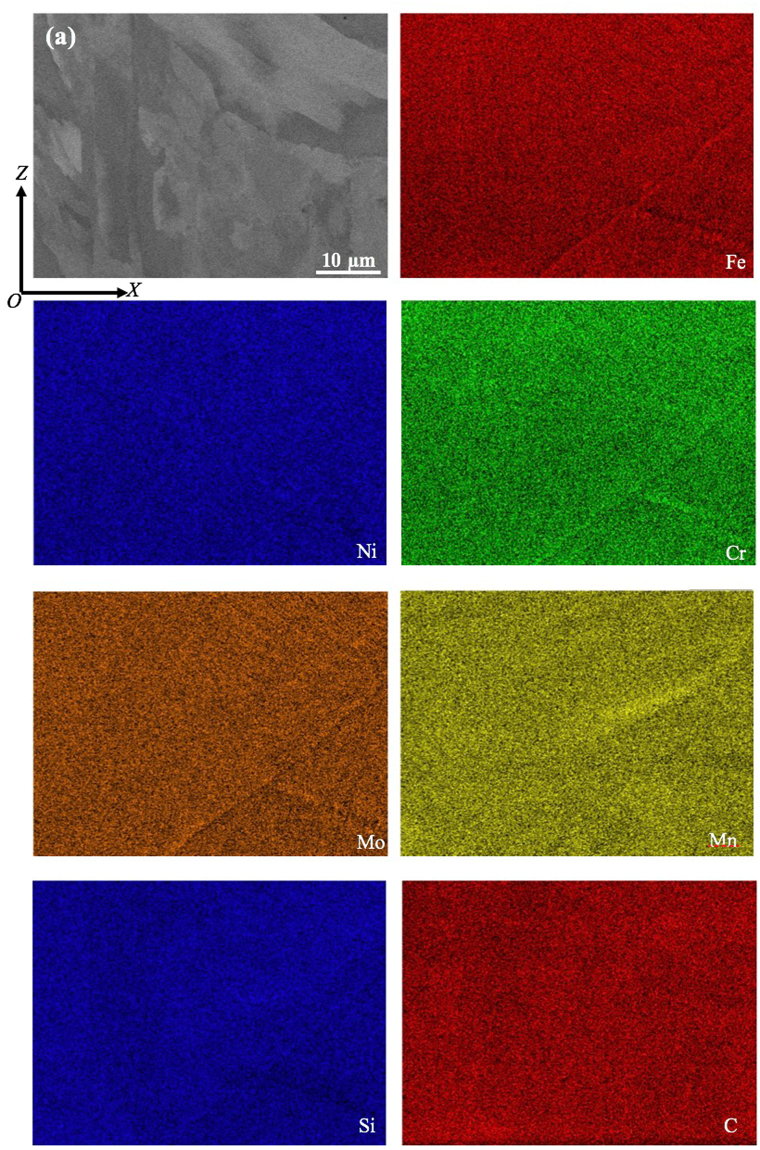
(**a**) Backscattered electron SEM image showing an area of an as-fabricated sample for EDX analysis and (**b**) EDX mapping results for various elements present in the sample.

**Figure 10:**
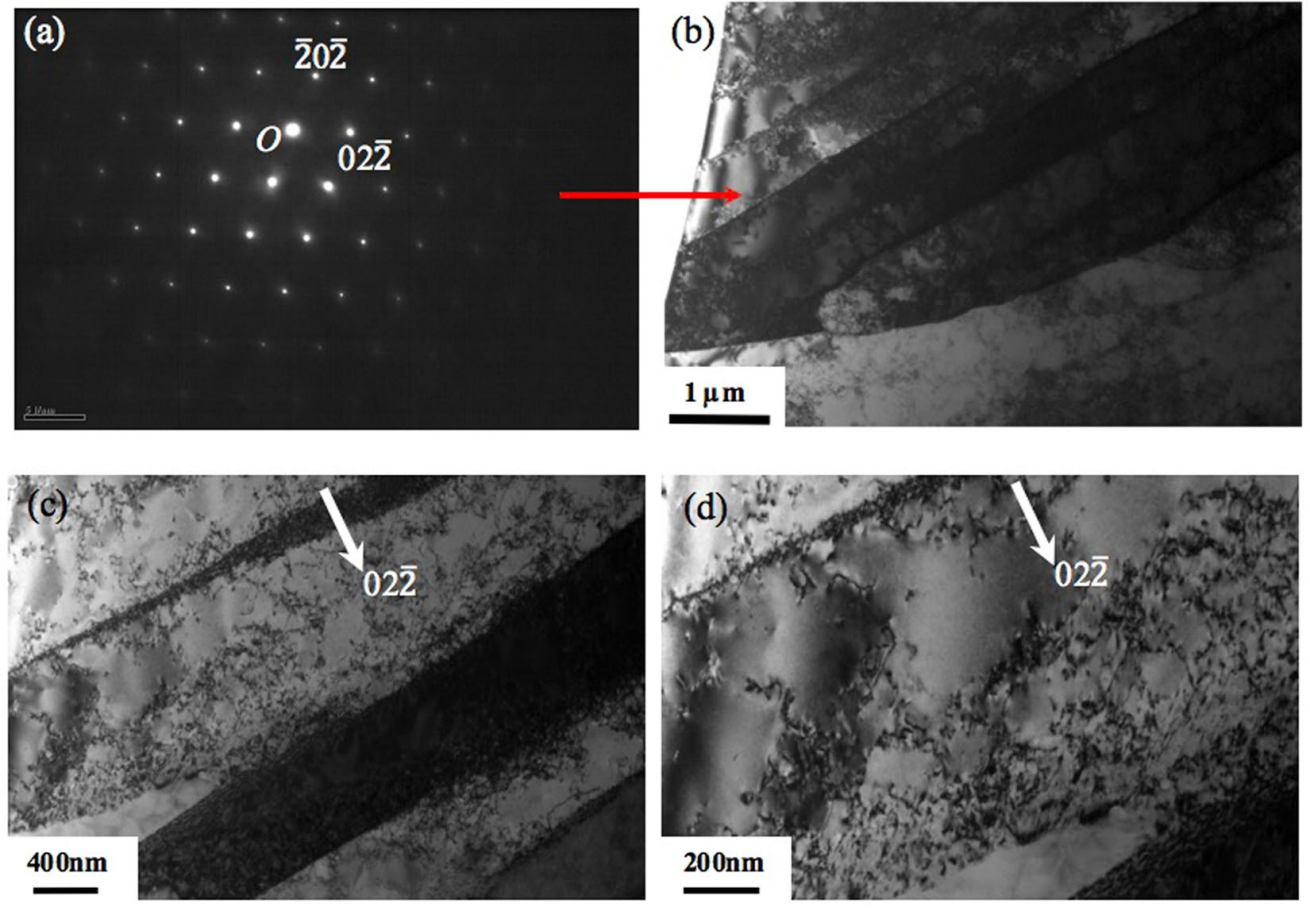
(**a**) A TEM SAD (selected area diffraction) pattern from a nano-needle in $[\bar{1}11]\,$ zone; (**b**) and (**c**,**d**) BF (bright field) TEM micrographs of a nano-needle taken from $[\bar{1}11]\,$ zone and under two beam condition, respectively.

**Figure 11:**
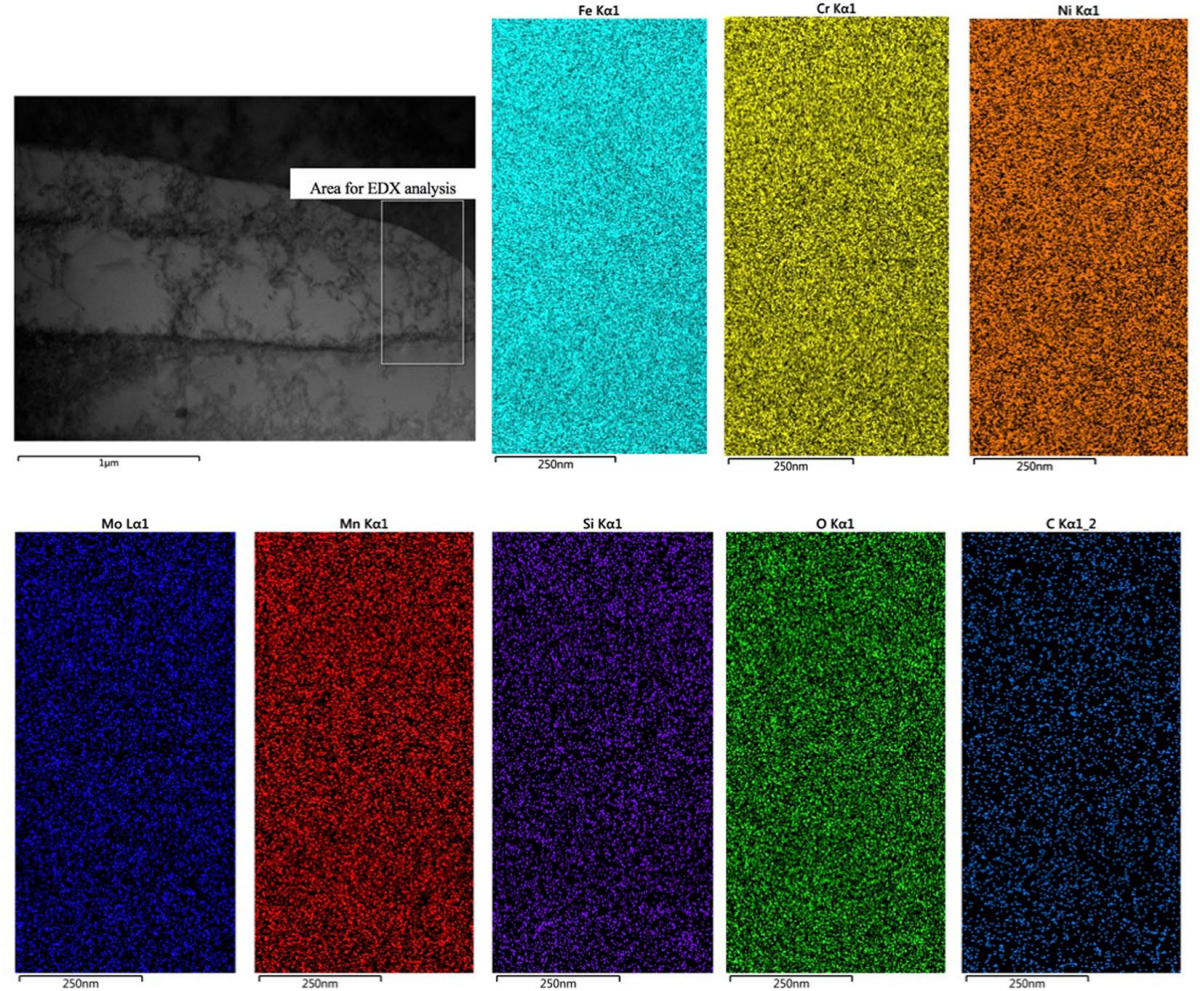
TEM-EDX mapping analysis results on an area containing parts from three neighbouring needles/cells and their interfaces.

Given that the formation of needle or cellular structure during solidification after SLM is usually associated with micro-segregation^6,7,12–14^, to understand the formation mechanism of nano-needle/cellular structure in the current samples, chemical analysis on the as-fabricated samples has been performed using EDX analysis technique in both SEM and TEM microscopes. Figure 9 shows the SEM-EDX analysis result on a relatively large area of a SLM-processed 316L sample. The results clearly indicate that the chemical distribution in the current sample is highly homogeneous. Given that the probe size for the current SEM-EDX analysis is around 1 µm, the result suggests that good chemical homogeneity has been achieved down to micron level. This result is in great contrast against many previous studies on SLM-processed 316L where SEM-EDX usually easily revealed segregation in as-fabricated 316L^12–14^. EDX in a TEM (beam size is smaller than 1 nm) was then further employed to study the structure and chemistry of the nano-needles at an even smaller scale, nanometre level. Figure 10 shows the TEM imaging results of a SLM-processed 316L sample. It is clear that a lot of dislocations are present both within nano-needles/cells and at the interfaces between them. Moreover, these dislocations tend to exist in a network form, which is consistent with Liu *et al*.’s work on SLM-processed 316LSS and is believed to play an important role in the tensile behaviour of this material^28^. These dislocations are believed to be mainly due to the development of internal stress and localised strain during rapid cooling after solidification in SLM process. TEM-EDX mapping on an area containing parts of two needles/cells and their interface shows that there is no micro- or nano-segregation at their interface (see Fig. 11), suggesting that the current SLM-processed 316L samples have a very good chemical homogeneity. This also means that the formation mechanism of nano-needles/cells in the current material is not necessarily associated with the development of micro-/nano-segregation at the interfaces between them.

**Figure 12:**
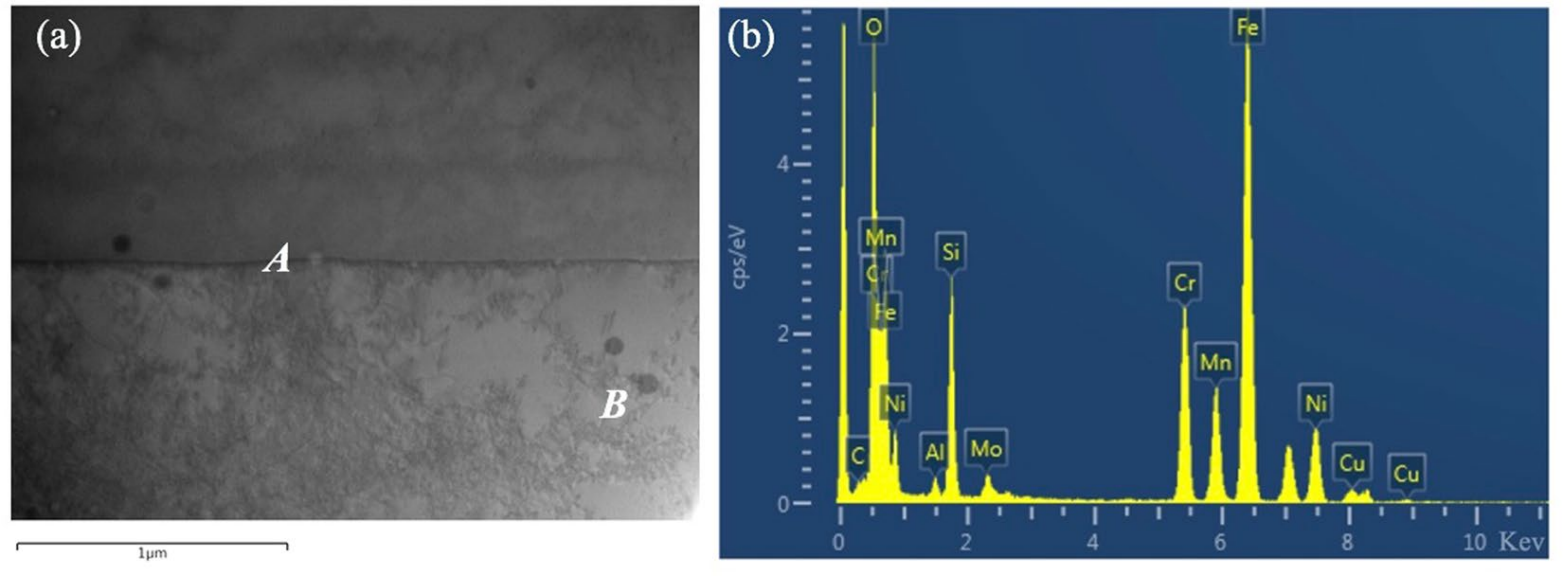
(**a**) Many beam TEM image of an area containing two cells, *A* and *B* correspond to two different precipitates; (**b**) EDX analysis spectrum on precipitate *B*.

It is also noted that there are some precipitates present in the current samples; see the TEM image in Fig. 12. TEM-EDX analysis (see Table 2) shows that these precipitates are generally enriched in Si, Mn and O as compared with the matrix regions and thus could be attributed to be (Si, Mn)O_2_. These precipitates tend to distribute randomly throughout the samples; most of them are within nano-needles and some are present at the interfaces between needles.

**Table 2:** TEM-EDX analysis results showing the compositions of a matrix area and some precipitates present in the current samples (wt.%).

Element	Matrix area	Precipitate A	Precipitate B
C	0.1	0.03	0.03
O	0.8	3.88	18.86
Si	0.8	2.81	9.41
P	0	0	0.02
S	0.1	0.09	0.44
Cr	19.8	19.73	14.73
Mn	1.4	5.44	7.43
Fe	63.1	54.49	40.42
Ni	11.2	9.72	7.14
Mo	2.8	3.81	1.51

### Investigation on tensile behaviour

**Figure 13:**
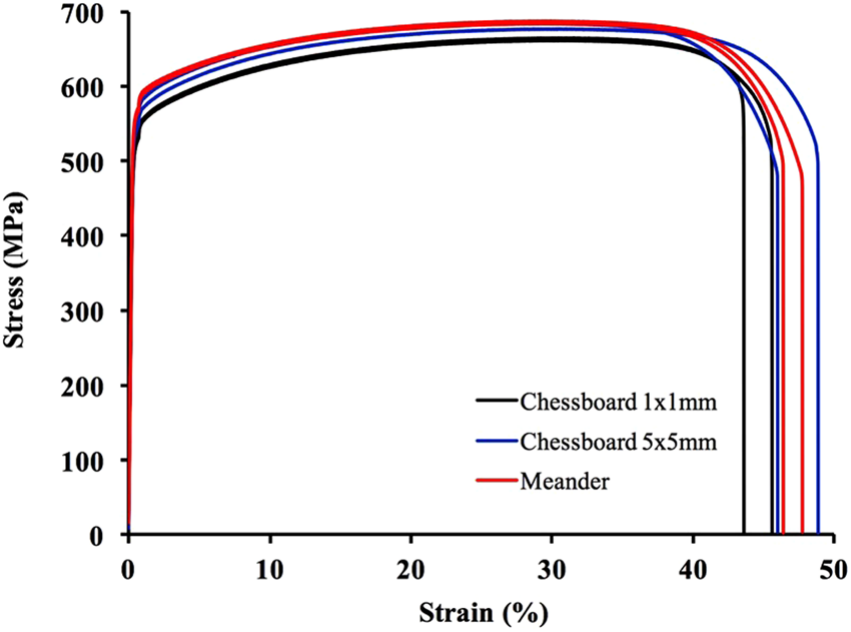
Tensile stress-strain curves for 316L stainless steel samples fabricated at 200 W and with different laser scanning strategies.

**Figure 14:**
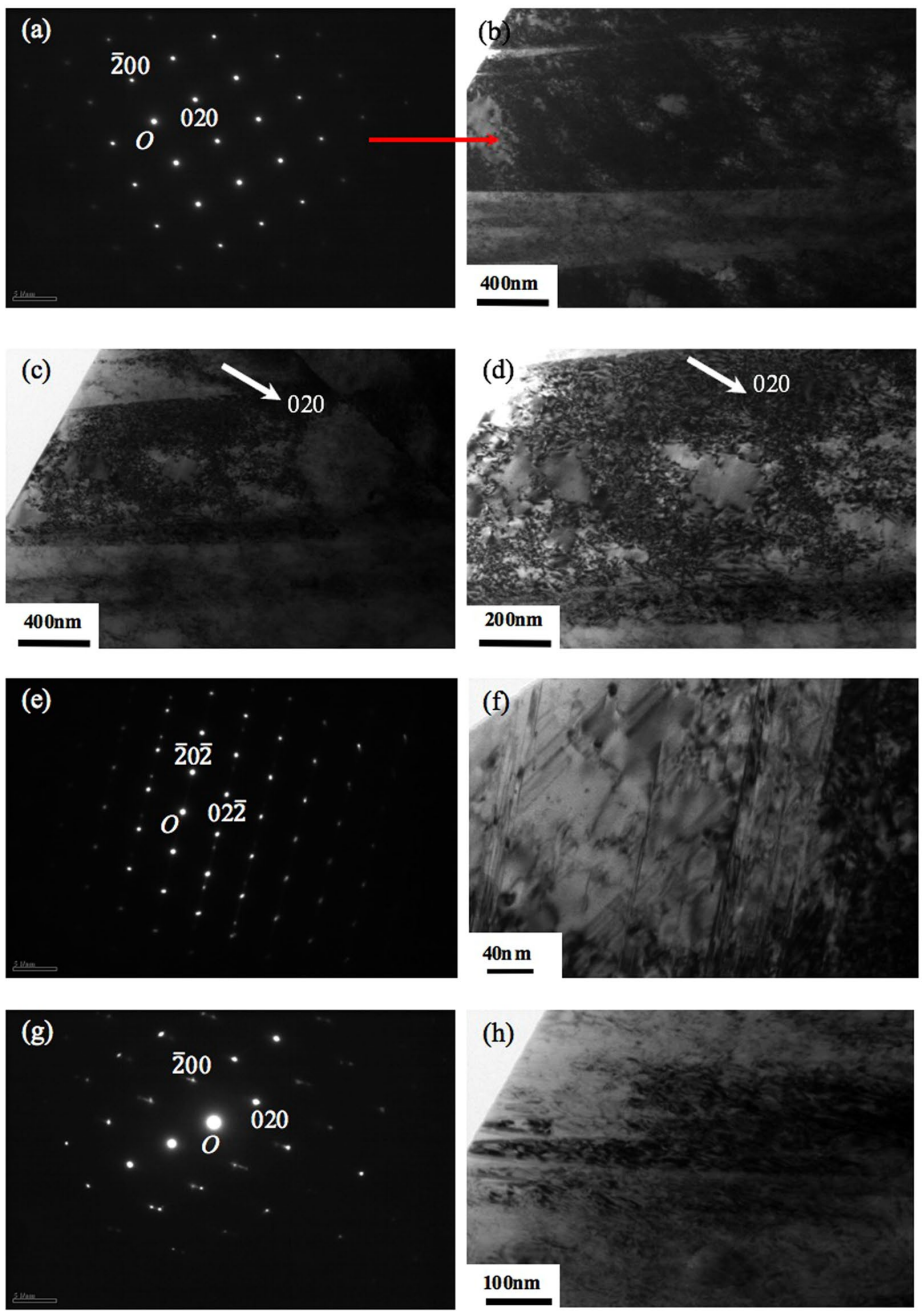
TEM analysis results of a SLM-processed 316L sample after tensile testing, (**a**) a SAD pattern from a nano-needle/cell in [001] zone; (**b**) and (**c**–**d**) BF TEM images of a nano-needle taken from [001] zone and under two beam condition, respectively; (**e**) and (**g**) SAD patterns from two different areas of the TEM specimen; (**f**) and (**h**) BF TEM images corresponding to diffraction patterns (**e**) from $[\bar{1}11]\,$ zone and (**g**) from [001] zone, respectively.

Figure 13 shows the tensile testing results of samples produced with different laser scanning strategies. All the samples show very high strength (0.2% yield strength > 500 MPa; UTS > 650 MPa) and good ductility (Elongation > 45%). There is no great difference in strength and ductility among samples fabricated with different laser scanning strategies, although the samples fabricated with Meander scanning strategy show marginally higher strengths than the rest of the samples. Among all the samples, samples made with Chessboard (1 × 1 mm islands) scanning strategy show the lowest strengths and elongations. Nonetheless, all the current samples show better strengths and ductility than conventionally manufactured 316L; see Table 3. Moreover, it is noted that there is some kind of scatter in tensile properties of SLM-processed 316L among different reports. Casati *et al*.^8^ reported comparable strengths but much lower elongations as compared with the current SLM-processed samples. Wang *et al*.^11^ reported slightly better strengths in their samples made by Concept Laser machine but lower elongations than the current SLM-processed samples. For the samples made by Fraunhofer machine, the strengths are lower than the current samples but the elongations are better. The reason for this is very complex; it could be due to different SLM machines used, or different powder with different qualities or different processing conditions used. However, indeed, SLM-processed 316L generally show better strengths, comparable or better elongations as compared with their conventionally manufactured counterparts. To understand the deformation and strengthening mechanisms, some of the tensile tested samples were investigated using TEM. The results are shown in Fig. 14. It can be seen that an extremely high density of dislocations has been developed in the nano-needle/cellular structures which is much higher than that in the as-fabricated state as shown in Fig. 10. Some of the dislocation networks that were observed before tensile deformation still remain in the tensile tested samples. Moreover, twinning has been frequently observed in a number of nano-needles throughout the samples, suggesting that deformation via twinning has occurred during tensile testing. Based on the current results, it is obvious that both slipping and twinning were operative during the tensile deformation.

**Table 3:** Tensile testing results for SLM-processed 316L stainless steel samples.

Sample	0.2% Yield strength (MPa)	Ultimate tensile strength (MPa)	Elongation (%)	Reduction of area (%)
Meander	555	684	50.7	70.5
	561	688	50.9	72.8
Chessboard 5 × 5 mm	546	684	49.5	72.3
	535	677	52.7	70.0
Chessboard 1 × 1 mm	519	664	45.3	55.4
	518	662	48.1	63.1
Casati *et al*.^8^ on SLM-processed 316L	554	685	36	—
Zhong *et al*.^9^ on SLM-processed 316L	487	594	49	58
Wang *et al*.^11^ on SLM-processed 316L (Concept Laser)	590	700	36	—
Wang *et al*.^11^ on SLM-processed 316 L (Fraunhofer)	450	640	59	—
Hot finished + annealed^29^	170	480	40	50
Cold finished + annealed^29^	170	480	30	40
Cold finished^29^	310	620	30	40

**Figure 15:**
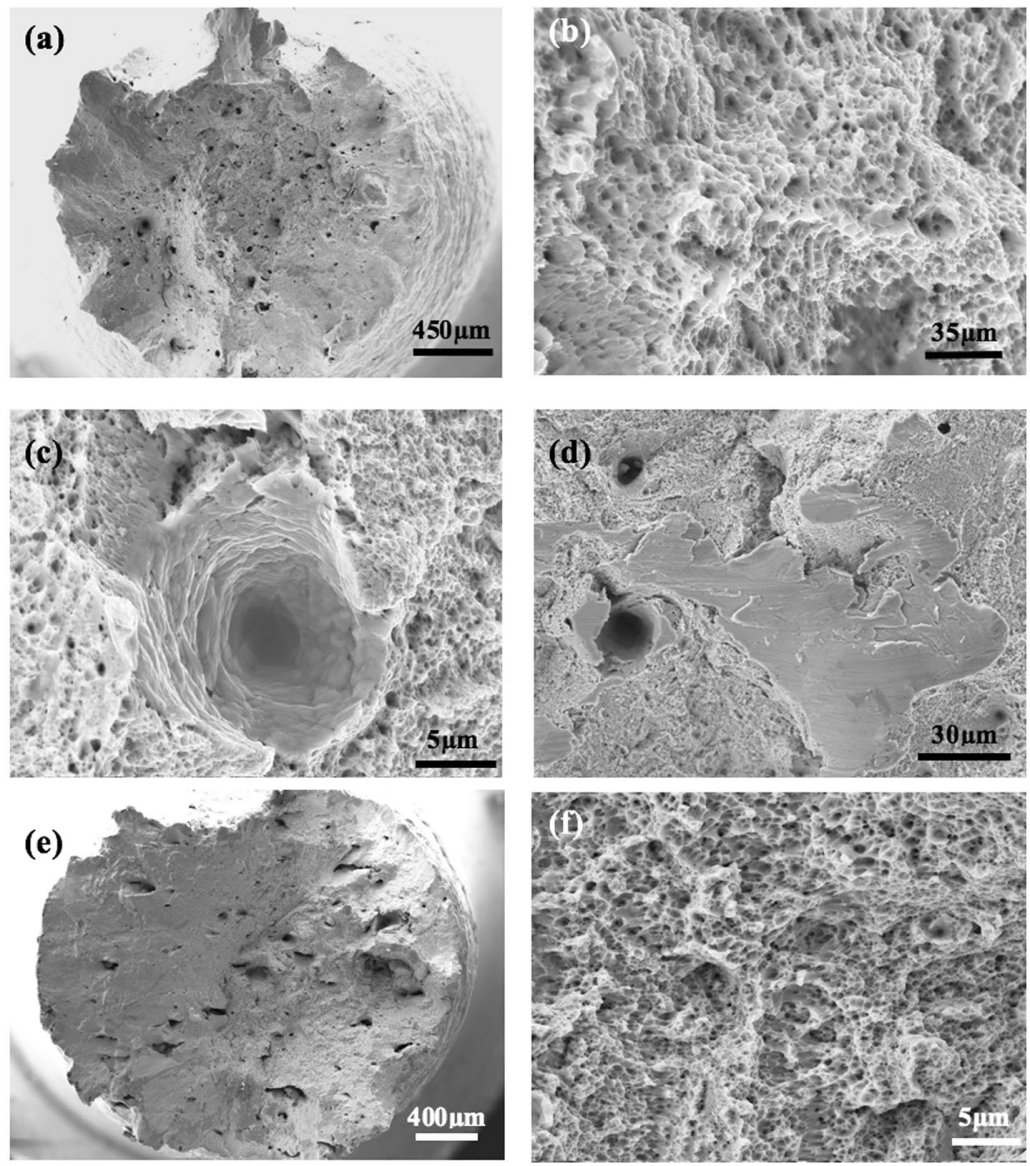
SEM Micrographs showing the fracture surfaces of samples fabricated at 200 W with different laser scanning strategies after tensile testing, (**a**–**d**) Meander; (**e**–**f**) Chessboard with 1 × 1 mm islands.

The fracture surfaces of the tensile tested samples were also examined using SEM and the results are shown in Fig. 15. It is clear that all these samples failed in a fairly ductile mode as indicated by the presence of a lot of fine dimples on the fracture surfaces, despite the presence of some voids in the samples.

## Discussion

The current experimental results clearly demonstrate that the as-fabricated 316L is dominated by γ nano-needle/cellular structure and its chemical distribution is highly homogeneous. There is even no micro-segregation at the interfaces between *γ* cells as evidenced by the TEM + EDX analysis, suggesting that the solute trapping effect has happened, i.e., the partitioning coefficient of solute elements (described as $\frac{{\rm{solute}}\,{\rm{concentrate}}\,{\rm{in}}\,{\rm{solid}}}{{\rm{solute}}\,{\rm{concentration}}\,{\rm{in}}\,{\rm{liquid}}}$) at the solid/liquid interface during solidification is equal or close to unit. This finding is different from previous studies on the same material where obvious segregation of Cr and Mo at cell walls has been observed^12–14^. It is known that solute redistribution during solidification of a material depends on its composition but is also greatly affected by solidification/cooling rate. Given that the same material has been used in the current and previous studies, the difference in solute redistribution should not be mainly due to slight compositional difference in the powders used. Instead, it is more associated with cooling rates. The current study applies a pulsed laser mode for SLM which is expected to give rise to more discrete molten pool (which can be evidenced by the development of more independent and random grain distribution within each weld bead) and thus to higher cooling rate after SLM than continuous laser mode. According to Elmer *et al*.^15,16^, solute redistribution during solidification of stainless steels is highly associated with cooling rate; with increased cooling rate, solute redistribution will be reduced. This is believed to account for the different observations on chemical distribution between the current study and previous studies where a continuous laser mode was used^12–14^. The cooling rate for the present study can be obtained by using the relationship between the dendritic arm space (DAS) and the cooling rate that has been developed experimentally by Katayama and Matsunawa for 316L stainless steels^17^

1 $$\lambda =80{\dot{{\rm T}}}^{-0.33}$$

where *λ* (μm) is primary DAS (in the current case, it refers to the spacing between nano-needles) and $\dot{T}$ is cooling rate (*K*/s). Based on measurement, the average interspacing of the current nano-needles is around 700 nm, thus the estimated cooling rate according to equation (1) will be 1.7 × 10^6^ *K*/s, which is higher than the calculated cooling rates for SLM using a continuous laser mode that are generally between 10^−3^ *K*/s and 10^−4^ *K*/s^10^. At such a high cooling rate, solidification should have been too rapid so that there is too little time for sufficient solute redistribution or diffusion to happen to generate obvious solute segregation. High cooling rate also leads to increase in solute partitioning coefficient and the chance of solute trapping effect (as demonstrated by the current results). Apart from the high cooling/solidification rate, the presence of O in the liquid of the current sample may have also played a role in avoiding segregation of ferrite solutes such as Si and Mn towards cell walls during solidification. According to the current TEM and EDX studies, O seems to have reacted with these elements during solidification to form Si and Mn oxides (see Fig. 12 and Table 2). These precipitates distribute randomly throughout the as-fabricated samples, suggesting that they were formed directly from fresh melts instead of from residual melts that remained after the formation of cells. However, given the low content of O, Mn and Si in the current material, the good chemical homogeneity in the current samples should still be mainly due to the extremely high cooling rate during SLM. To fully understand this issue, mathematical modelling on the solute redistribution and solidification behaviour after SLM is necessary and will be part of our future work.

The current experimental results also demonstrate that with the presence of γ nano-needle structure, the SLM-processed 316L shows superior strengths and ductility as compared with conventionally manufactured counterparts. Pham *et al*.^27^ attributes the superior ductility of SLM-processed 316L to twinning deformation and suggests that twinning does not contribute to the high strength. The role of twinning in both deformation and strengthening of SLM-processed 316L is recognised by Wang *et al*.’s work^12^. In the current study, the TEM analysis on tensile tested samples reveals both an increased density of dislocations and a number of twinning in the needle structures of the current samples after tensile deformation, indicating that both dislocation slipping and twinning have been activated during deformation. It is thus suggested that the superior ductility of the current samples should be due to both gliding of dislocations and twinning deformation. The superior strength is believed to mainly originate from the presence of residual stress in the as-fabricated samples (evidenced by the presence of a high population of dislocation densities) and work hardening induced by the development of significantly high density of dislocations within the nano-needles/cells and the cell walls during deformation. Moreover, it is noted that a lot of dislocations exist in network form in the as-fabricated 316L samples and many of these dislocation networks remain after tensile deformation. This is consistent with Liu *et al*.’s work^28^ where similar observation has been made. They further suggest that the presence of dislocation networks in the SLM-processed 316  LSS is able to slow down but not entirely block the dislocation motion and also promote the formation of a high density of nano-twins during plastic deformation, both contributing to the improvement of tensile strengths and ductility. It is obvious that the unique γ nano-needle structures together with the dislocation structure within them are crucial for the acquisition of superior tensile properties in SLM-processed 316L.

Finally, the current experimental results also demonstrate that laser power plays a more dominant role in porosity and grain development during SLM than laser scanning pattern. These results suggest that the local input power density (which is more associated with laser processing conditions such as laser power and scanning speed) is more important than the overall heat distribution on a single cross section (which is associated with the laser scanning strategy) in porosity and grain development during SLM. The findings are of great engineering value to industry as parametric study is a time- and labour-consuming process and identification of some of the most important parameters is useful for industrial scale production.

## Conclusions

- Selectively laser melted 316L samples were characterised by columnar grains which are dominated by clusters of *γ* nano-needles/cells oriented in different directions.
- The formation of *γ* nano-needle/cellular structure is not associated with development of segregation at interfaces between them, which is attributed to the extremely high cooling rate after the current SLM process and to the formation of Si- and Mn-oxides that randomly distribute throughout the samples.
- With the presence of *γ* nano-needle structure, the SLM-processed 316L stainless steel samples show superior strengths and ductility as compared with conventionally manufactured counterparts.
- The plastic deformation of *γ* nano-needle/cell structure was governed by both slipping and twinning deformation.
- Laser power plays a more dominant role than laser scanning pattern in porosity and grain structure development for selectively laser melted 316L.

## Methods

**Figure 16:**
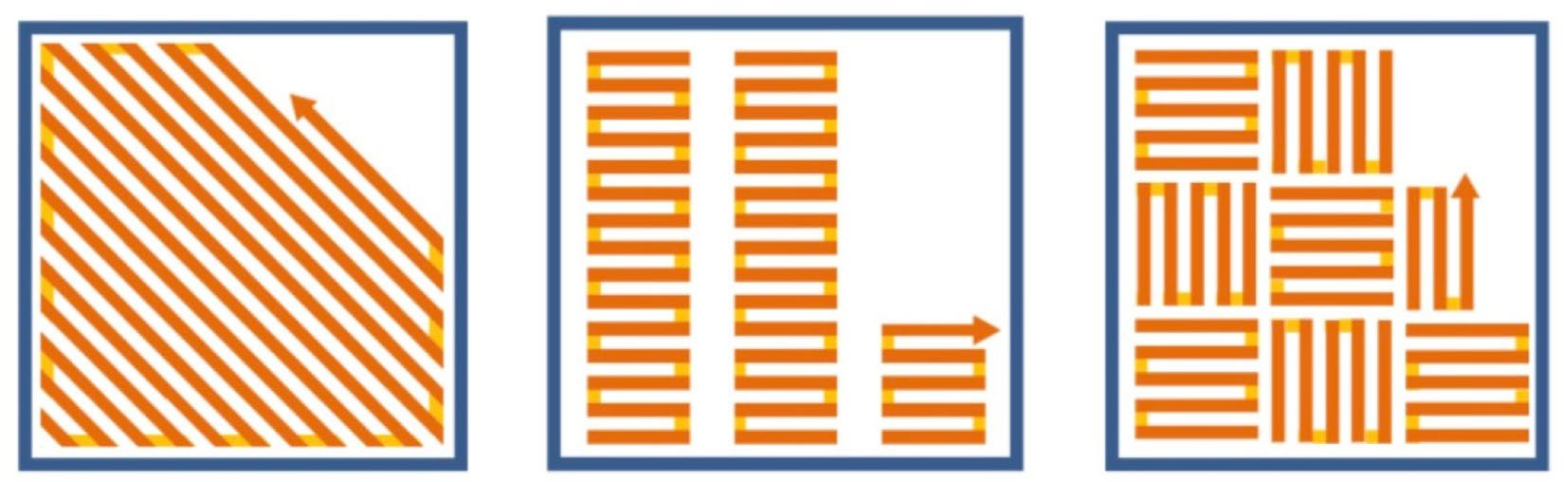
Schematic illustration showing the laser scanning strategies used in the current study, (**a**) Meander; (**b**) Stripes; (**c**) Chessboard.

The material used in this study was gas atomised 316L powder supplied by Renishaw plc in the size range of 15–45 µm. A Renishaw 250 SLM system which employs a fiber laser with 200 W maximum power was used to prepare samples with a dimension of 10 × 10 × 12 mm for microstructural characterization and samples with a dimension of 60 × 10 × 10 mm for tensile testing. Samples were fabricated in argon with different laser scanning strategies and at different laser powers such as 110 W, 140 W, 170 W and 200 W under argon atmosphere. The laser scanning strategies used in the current study include Meander, Stripe and Chessboard scanning strategies which are illustrated in Fig. 16. Among all the scanning strategies, Chessboard scanning strategy divides a cross section into a number of square islands which are then randomly scanned afterwards. Two island sizes 1 × 1 mm and 5 × 5 mm have been tried in this study. The stripe width for the Stripe scanning strategy was set up to 5 mm. Once the hatch scanning is completed on a cross section, a contour scan will be performed around the edges of the section. The distance between the hatch boundary and the contour was set to be 60 µm. The scanning direction was rotated by 67° after each layer, therefore, the same pattern was repeated every 180 layers. This is believed to be helpful in removing defects such as porosity created in previous layer.

A pulsed laser mode was used in the current study where the laser operated by using point exposures instead of running continuously. This is believed to be able to generate more discrete melt pools and thus even higher cooling rate after SLM than the continuous laser mode. The laser beam diameter used was around 75 µm and the exposure time for each pulse was 80 µs. Laser melting was performed by radiating discrete and partially overlapped laser spots. The spacing between the central points of two neighboring laser spots or melt pools was set to be 60 µm. At the end of each scan line, the laser jumped to a partially overlapped adjacent track. The spacing between central lines of two neighboring laser scanning tracks (or hatch distance) was defined to be 110 µm. The thickness of each powder layer processed in the current study was 50 µm and the laser scanning speed (i.e. the speed of laser moving from one exposure to another) was 5000 mm/s.

The as-fabricated samples were ground using SiC papers from 200 grits up to 4000 grits before being polished using 3 µm diamond suspension and then colloidal silica suspension (or OPS solution). The samples were then electrolytically etched in a solution which contains 10 g Oxalic acid and 100 mL water prior to microstructural characterization using OM and SEM. X-ray diffraction (XRD) was performed on some of the as-polished samples with a Phillips diffractometer using Co K𝛼 radiation with a wavelength of 0.1789 nm. An FEI XL30 Field Emission Gun Environmental SEM (ESEM) has been used to characterize the microstructure of the as-fabricated samples and a Zeiss Sigma HD Field Emission Gun Analytical SEM (ASEM) fitted with two Oxford Instruments 150 mm^2^ EDX used to conduct X-ray elemental mapping to investigate the chemical distribution within the current samples. EBSD was performed on some of the as-polished samples in a Merlin Zeiss SEM to investigate the grain structure. TEM study was also performed on the as-fabricated samples. Disc specimens with 3 mm in diameter were machined out of the as-fabricated samples and ground into a thickness of 150–200 μm using 400–800 grade silicon carbide paper and then electro-polished to perforation using a twin-jet electropolisher (StruersTenupol-5). A polishing solution containing 5% perchloric acid and 95% ethanol was used to electro-polish the samples. TEM imaging was carried out with an accelerating voltage of 200KV in an FEI TecnaiF20 FEG TEM microscope while TEM-EDX was conducted in a JEM-2010F TEM microscope.

For tensile testing, the as-fabricated elongated samples were machined into cylindrical specimens with a parallel length of 19 mm and a nominal diameter of 4 mm. Tensile tests were performed at room temperature following the ASTM E8 standard method. The specimens were tested in strain rate control at 10^−4^/s to beyond yield at which point another strain rate of 8.33 × 10^−4^/s was adopted until failure. After tensile testing, the fracture surfaces of specimens were examined using SEM and the internal structure of the tested samples examined using TEM.

### Data Availability

The datasets generated during and/or analysed during the current study are available from the corresponding author on reasonable request.
